# Microplastics negatively impact ecosystems, human health and cellular proteostasis

**DOI:** 10.1098/rstb.2025.0268

**Published:** 2026-10-01

**Authors:** Elise Kikis

**Affiliations:** Department of Biology, The University of the South, Sewanee, TN 37383, USA

**Keywords:** microplastics, protein folding, proteostasis

## Abstract

With the widespread use of plastics for both personal and industrial applications, significant amounts of micro- and nano-sized plastic particles (MNPs) are entering our air and waterways. This impacts ecosystems, triggers the formation of biofilms and facilitates the emergence of antibiotic-resistant bacteria. Moreover, direct human exposure to MNPS is inevitable and occurs primarily through ingestion and inhalation. Once internalized, MNPs cause oxidative stress and inflammation. Within tissue matrices, proteins and other macromolecules adhere to MNPs, forming a coating referred to as a corona. This corona provides the MNPs with biological identity that increases their propensity to enter cells and breach the blood–brain barrier. From a proteostasis perspective, proteins that physically interact with MNPs undergo conformational changes that result in the formation of a rigid β-sheet structure. This leads to amyloidogenesis in a manner that may pose a risk for neurodegenerative disease. This review synthesizes what is known about the properties of microplastics (MPs), their ecological impacts and their impacts on human health—with an emphasis on the ways that MP may interfere with the ability of cells and organisms to maintain healthy proteostasis.

This article is part of the Theo Murphy meeting issue ‘ProteostaSys: a systems view of proteostasis'.

## 1. Introduction

### (a) What are microplastics?

Plastic, in its ‘macro’ form, is ubiquitous. It takes on various forms—food containers, grocery bags, electronic equipment, furniture, clothing, toys, paint and much more. Although we may not always realize it, we regularly come into contact with plastic beads that were intentionally manufactured to be micro-sized (less than 5 mm in diameter). These exist as microbeads in personal-care products (facial cleansers, toothpaste) and in the form of ‘nurdles', which are pellets of industrial resin used to manufacture plastic products. However, it has not always been this way. Plastic is a modern invention. The first completely synthetic plastic was invented in 1907, but it was not until the late 1920s that modern plastics such as polyvinyl chloride (PVC) and polystyrene (PS) were developed, with commercialization starting in earnest less than 100 years ago. At around the time of the Second World War, the ‘plastic age’ began. Plastic is lightweight, durable, flexible and highly amenable to mass production. Unsurprisingly, plastic quickly became the material of choice for much of the manufacturing sector, forever altering the way that people interact with, and pollute, the environment.

Currently, the world produces as much as 350 million tons of plastic each year. Much of this material enters the environment as plastic refuse, with degradation rates ranging from 100 to 1000 years. Thus, although plastic is no doubt a revolutionary material in terms of manufacturing, its ubiquity has led to an environmental crisis whose impacts we are only starting to comprehend [1–4]. This crisis is exacerbated by the fact that only a small fraction of plastic waste is recycled or incinerated, with the vast majority making its way to landfills or directly into the natural environment [5,6].

The biological impacts of plastic on ecosystems and organisms depend on various factors, including size, with the smallest plastic particles being the most toxic. Microplastics (MPs) are defined as particles that are between 1 and 5 µm in diameter, and nanoplastics (NPs) are generally <1 µm. Micro- and nano-sized plastic particles (MNPs) are classified, based on their origin, as either ‘primary’ or ‘secondary’ [1–3]. Primary MNPs are intentionally manufactured for commercial applications, such as the microbeads found in personal care products. Secondary MNPs are formed unintentionally when larger plastic items fragment and degrade in the environment [1–3,7].

Plastic degradation occurs through physical, chemical and biological processes. Physical forces such as pressure and thermal expansion can cause fragmentation, whereas mechanical abrasion (the repeated grinding or rubbing of materials) is a distinct form of physical degradation that plays a major role in MNP generation [7]. Common examples of mechanical abrasion include the erosion of car tyres against road surfaces [8] and the release of synthetic fibres from textiles [9]. Chemical degradation, particularly ultraviolet (UV)-mediated photoageing (the breakdown of polymers caused by UV light), further contributes to fragmentation [10]. Finally, enzymatic activity carried out by microorganisms has been shown to alter and weaken plastic structures, facilitating their breakdown into smaller micro- and nano-sized particles [11,12].

### (b) Human contact with nano-sized plastic particles

Polyethylene (PE) is the most widely produced synthetic polymer. It is used to manufacture packaging, household products and construction materials. It can be engineered to be either flexible or rigid, making it especially versatile. A study of the degradation of PE revealed that a combination of UV-C irradiation and mechanical stress, performed in a controlled laboratory environment, resulted in progressive structural breakdown [4]. Specifically, MP fragments were found to be shed from the primary PE plastic and to subsequently break down further into NPs. Interestingly, NPs were also shown to be shed directly from large pieces of intact PE, suggesting that NPs can enter the environment quickly and easily [4]. This also implies that we may be coming into contact with MNPs more readily than previously assumed. For example, pre-packaged non-alcoholic beverages were all found to contain measurable amounts of MPs [13]. This was true even in beverages packaged in non-plastic containers [13]. PS was the most abundant plastic type found in pre-packaged beverages [13]. The authors of the study posited that the most probable source of the MPs found in packaged beverages is ‘atmospheric fallout’—the introduction of MPs during the process of filling containers. Additionally, the packaging itself is also a likely source of MP contamination in these products, probably as a result of the mechanical stress that is placed on plastic bottles during capping, sealing and transit [13]. This is consistent with PS being the most abundant MP identified in this study, as PS is typically used to manufacture plastic screw caps for bottles.

Another manner by which we come into contact with synthetic polymers is via tyre wear products that make their way into the air and waterways. They also compose flooring material for playgrounds and synthetic sports turf, both of which are often intentionally manufactured from recycled rubber tyres. Modern tyres are composites of many materials, including synthetic rubber (a petroleum-based polymer) and synthetic textiles such as polyester, nylon and rayon. To harden the tyre material, it is vulcanized using sulfur and other materials that prevent degradation by heat and oxygen. Tyres consequently pose such significant environmental hazards that they cannot be disposed of in landfill. Thus, they are generally repurposed into fuel or ground down into ‘crumb rubber’.

Crumb rubber represents approximately 38% of all the MP that is released into the environment. As noted above, it is a common flooring material in playgrounds and is incorporated into synthetic athletic turf. The European Union has taken steps to ban crumb rubber from playgrounds and sports turf on account of its containing toxic heavy metals [14]. Likewise, several major cities in the USA, including Washington DC, San Francisco, Los Angeles and New York City, have prohibited or curtailed its use [15]. Nonetheless, crumb rubber continues to be a material of choice for flooring and turf, especially because it is generally considered to be an environmentally conscious way to repurpose old and discarded rubber tyres. However, crumb rubber contains large amounts of metals and metalloid compounds that can be toxic [16]. Thus, its use on playgrounds and sports turf, where people, especially children, come into close contact with it, is concerning. Crumb rubber samples from playgrounds and sports turf have especially high concentrations of zinc, aluminium and iron [16]. Lead was reported to exceed levels approved in the EU for toy material. Moreover, other heavy and toxic elements were identified, including chromium, arsenic and cadmium [16].

Another way in which we come into physical contact with MP particles, and introduce them into the environment, is via synthetic textiles, such as polyester, polyamide (nylon), acetate, acrylic, polypropylene and rayon. The manufacturing process for these textiles produces a great deal of MP fibres (MPFs), much of which are released into the air and water. Additionally, MPFs have the tendency to be shed from the resultant clothing. These fibres contact the skin of the wearer and also are released into the air. They enter waterways via the wastewater produced by the machine-washing of synthetic textiles, thereby making their way into aquatic ecosystems. In a study aimed to determine factors that contribute to microfibre release via machine-washing, the researchers examined the weight of the textiles and/or their construction (knitted or woven). They found that woven acrylic released the largest amount of MPFs during the machine-washing process, recycled polyester released more MPFs than non-recycled, and the pre-wash cycle, optional on many household washing machines, greatly increased the extent of MPF release [17].

### (c) Toxicity of microplastics and environmental impacts

When MPFs and other MPs are released into wastewater, they have the potential to significantly impact aquatic life. For that reason, much of the effort to study their environmental impacts has been focused on aquatic organisms and aquatic ecosystems. Wild isolates of *Chlorella pyrendoisa*, a species of microalgae that serves as a primary producer in freshwater ecosystems, were exposed to MPFs in a laboratory setting. Compared with natural fibres such as cotton and wool, MPFs caused oxidative stress and inhibited growth [18]. Strikingly, the toxic effects of MPF exposure were markedly greater even than those of asbestos exposure, which served as the positive control for toxicity [18].

Cladocerans are tiny aquatic crustaceans less than 5 mm in diameter. Like several animals in this superorder, *Daphnia magna* serves as an important toxicological model organism. When *D. magna* were chronically exposed (54 days) to primary MPs, they experienced a dose-dependent decrease in the total number of individuals in a laboratory population. Moreover, exposure to MPs resulted in an overall decrease in the size of the exposed animals compared with their unexposed counterparts [19]. A study comparing the effects of pristine MP particles with those of secondary MP that were artificially weathered revealed that different species of cladocerans experienced a dose-dependent decrease in reproduction, with pristine MP being most detrimental. Species-specific differences in response to MPs were observed, with *D. magna* being especially sensitive to both types of MP and *Daphnia pulex* only experiencing a decrease in brood size upon exposure to pristine MP [20].

MPF exposure may also affect the ability of aquatic animals to tolerate secondary stress. For example, MPFs were shown to be non-lethal on their own, but to enhance the susceptibility of rainbow trout (*Oncorhynchus mykiss*) to viral infection. When the fish were exposed to virus alone, they experienced an approximately 20% mortality rate. However, that number increased threefold when otherwise non-lethal amounts of MPFs were also present [21]. This suggests that MPs may compromise the tissues of host animals, which, in turn, could lead to an increased viral load [21].

### (d) Plastisphere

MPs can serve as substrate for biofilm formation. Such biofilms, often termed the ‘plastisphere’, have been proposed as a strategy to accelerate the degradation of plastic waste in a natural, biological, way [22]. However, if certain bacterial species are especially prone to form MP-associated biofilms, then, as MPs are introduced into aquatic ecosystems, we would expect to observe a shift in the natural bacterial composition. To test this, 16S rRNAs were analysed to ascertain the bacterial composition of biofilms associated with different types of MPs [23]. The results suggested the occurrence of species sorting. In other words, the biodiversity of MP-associated biofilms was found to be less than that of the surrounding environment [23]. Specifically, Actinobacteria were less probable to form MP-associated biofilms than Alphaproteobacteria [23]. Similar findings regarding the decreased biodiversity of the plastisphere were obtained via a separate study of four different wetland ecosystems [24]. High-throughput qPCR was utilized to determine the relative expression of genes involved in carbon degradation, carbon fixation and denitrification, with several of these biogeochemical cycling genes being reported as upregulated in the plastisphere relative to the surrounding soil [24]. These findings are consistent with MNP-associated biofilms serving as hosts to distinct bacterial communities in a manner that could, over time, significantly alter the bacterial composition of aquatic ecosystems.

By bringing bacteria in close proximity to one another, the plastisphere may also facilitate the spread of antibiotic resistance genes through horizontal gene transfer (HGT). Several studies have examined this possibility, showing that the plastisphere is enriched in antibiotic resistance genes [25–27]. Although some studies have found that PVC may be a particularly good plastisphere substrate for HGT [26], others found that all MPs are equal in this regard [25]. Nonetheless, wastewater, which is rife with human pathogens, antibiotics and MPs of various origins, may be an especially potent incubator of antibiotic-resistant bacteria [25].

### (e) Health impacts

That MPs promote the emergence of antibiotic-resistant bacteria is concerning in terms of its obvious impacts on human health. Additionally, the human body can be harmed in myriad ways by direct contact with MNPs. Because MNPs are distributed in air, water and tissue matrices, human exposure is inevitable. The primary modes of entry are inhalation from the air [28,29] and the consumption of contaminated foods and beverages, including drinking water, aquatic products such as fish and crustaceans, honey and salt [30]. Owing to their small size, MNPs can penetrate physiological barriers and accumulate in many organs of the body, including the liver and kidneys [31]. In the gut, MNPs activate macrophages, triggering an inflammatory response that, via the gut–brain axis, leads to neuroinflammation and decreased cognitive function in mice [32]. This was even true of so-called ‘eco-friendly’ biodegradable plastic [33]. A comparison of the effects of MPs on macrophages, enterocytes, hepatocytes and alveolar epithelial cells revealed that weathered Styrofoam was especially toxic to macrophages, triggering an inflammatory response in a manner that was consistent with oxidative stress [34]. The size of the MNPs seems to greatly influence their ability to be internalized and their subsequent toxicity [35]. Specifically, fluorescently labelled PS particles, less than 0.5 µm in diameter, were readily taken up by macrophages, compared with larger particles, and were more likely to generate reactive oxygen species via mitochondrial damage [35]. Moreover, small (<0.5 µm) PS particles, administered orally or intravenously to mice, accumulated in organs and caused systemic toxicity that manifested as weight loss and oxidative stress [35]. This is consistent with findings in *Caenorhabditis elegans*, where oxidative stress and neurotoxicity were observed following exposure to material derived from pristine or artificially weathered plastic bowls [36].

NPs can even bypass the blood–brain barrier (BBB), probably via nasal inhalation pathways, to deposit directly in the brain [31], with astrocytes being particularly prone to accumulate PS MNPs [37]. Likewise, certain plastic types, including PS, polyethylene (PE), polypropylene (PP) and PVC, have been identified in human cerebrospinal fluid [38]. Once internalized, the MNPs seem to challenge cellular quality control and to exacerbate age-related neurodegenerative diseases [31,38,39]. Specifically, post-mortem brain tissue from dementia patients had significantly more MNP deposits than those of individuals without dementia [31]. Similarly, the amount of PE and PVC in Alzheimer's disease patients is directly correlated with amyloid plaques [38], which is consistent also with findings in mice [39]. These findings could be explained by MNPs triggering protein misfolding and contributing to age-related and disease-associated proteostasis collapse.

## 2. The nanoplastic–protein corona interface

### (a) Protein corona formation

Upon contact with biological fluids such as blood plasma, NPs rapidly acquire what is referred to as a protein corona—a layered coating of proteins and other biological macromolecules. This corona, comprising naturally occurring molecules, provides the NPs with a sort of biological identity that has the ability to dictate subsequent cellular interactions, including NP internalization via endocytosis [40,41]. Corona formation is primarily driven by the hydrophobic nature of the plastic surface, which is thus able to interact with hydrophobic patches on proteins [40,41] (figure 1B). Initially, proteins form weak associations with NPs and make what is known as a *soft corona*. This is a dynamic state that shifts over time to a more stable, tightly bound, inner layer of proteins referred to as the *hard corona*. The hard corona is characterized by definitive structural alterations in the adsorbed proteins [42]. The critical event in the establishment of a hard corona is an NP-induced helix-to-β-sheet conversion. Specifically, studies show that when binding to nano-sized PS particles (nPS), human serum albumin (HSA) and ubiquitin (Ubq) lose α-helix content and gain a stoichiometric amount of β-sheet structure [43,44].

**Figure 1.**
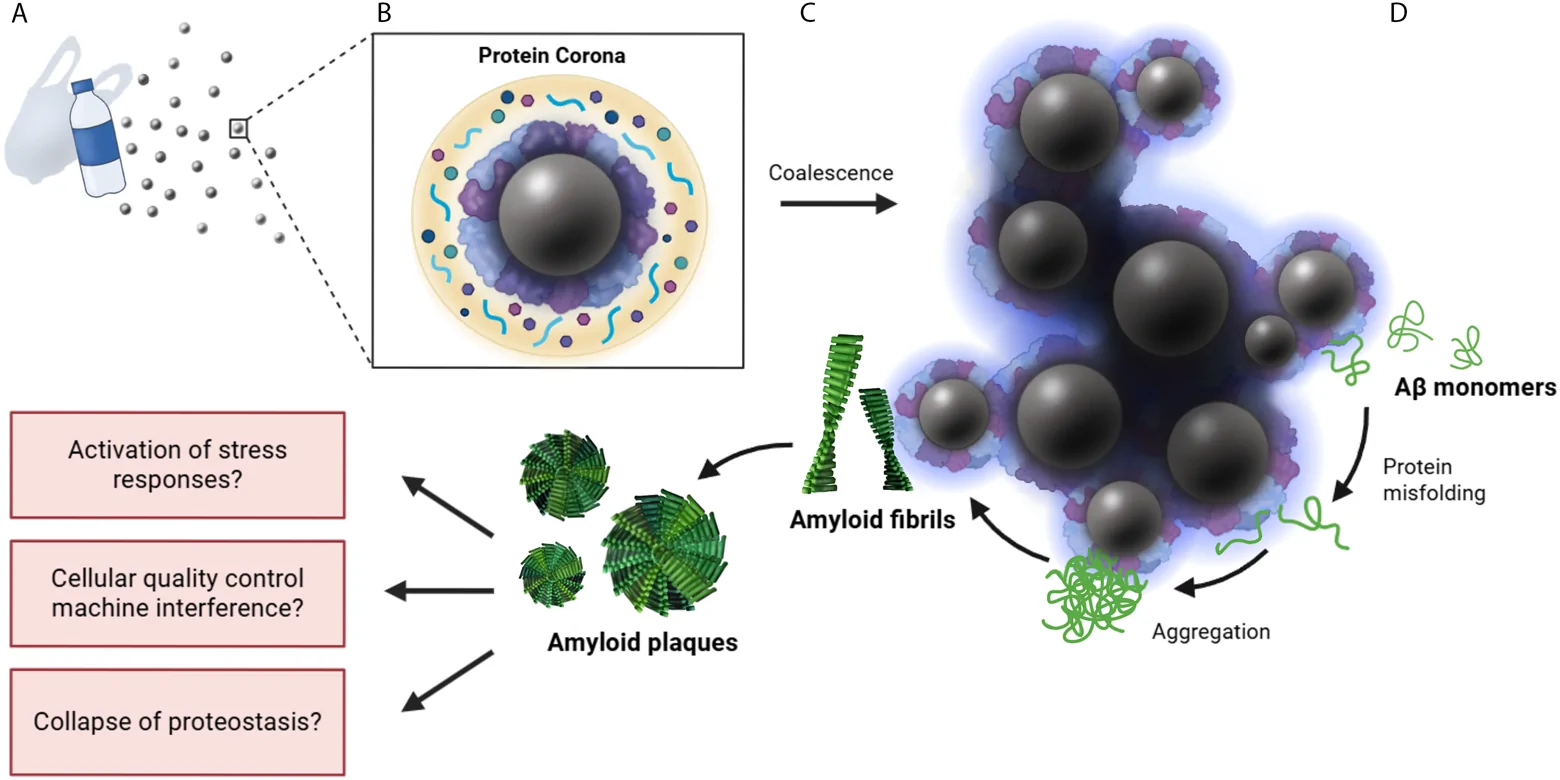
Nano-sized plastic particle (MNP) corona formation and its effects on protein folding. (A) MNPs enter the environment in a variety of ways, including the breakdown of large plastic products. (B) Upon interacting with biological systems, MNPs acquire a corona of proteins and other biological macromolecules. (C) Coronated MNPs coalesce into large particles, often via the interaction between components of the corona. (D) Soluble proteins, including amyloid beta (A β) monomers, interact with the coronated surface and undergo conformational changes that lead to amyloidogenesis. Together, the literature suggests that MNPs may activate stress responses, interfere with the quality-control machinery and/or contribute to proteostasis collapse.

### (b) Corona-mediated particle coalescence

In a process termed corona-mediated particle coalescence, the denatured or misfolded proteins in the corona have the tendency to self-associate. This causes coronated NPs to bridge together, with misfolded proteins acting as secondary binders. For example, apolipoprotein A-1 (ApoA-1) forms fibrillar β-sheet structures at the NP interface and aggregates upon contact with nPS [45]. The resultant bridges, formed by non-specific protein–protein interactions, link coronated particles and result in aggregates of coalesced coronated NPs that achieve diameters exceeding 1 µm (figure 1C). MNPs as large as these have been shown to be taken up by macrophages and impair their ability to phagocytose and kill *Escherichia coli* [46]. Such MNP-mediated disruption of macrophage function would be expected to impair the ability of macrophages to clear pathological proteins, such as the Alzheimer's disease-associated amyloid-beta (A β) [47].

### (c) Toxicity

Coalesced NPs, encased in coronas of structurally altered protein (figure 1C), exhibit heightened cytotoxicity compared with pristine (non-coronated) forms of plastic. Specifically, in blood, coronated NPs adhere to erythrocytes, triggering haemolysis and/or vascular blockage [44]. Moreover, in their coronated form, NPs achieve the ability to breach the BBB and bioaccumulate in the brain [31]. This leads to neuronal damage [36], microglial activation [32] and the disruption of metabolism [48]. Finally, coalesced NPs have been shown to disrupt the DNA repair pathways, trigger oxidative stress and induce protein misfolding [49,50]. Consistent with NPs representing a major neurotoxicity risk [31,51], their accumulation in the brain is reported to be toxic to mice [29] and to be significantly elevated in dementia patients [31], as described above. Again, these findings support the hypothesis that a major aspect in the mechanism of MNP toxicity is their effect on protein folding.

## 3. Protein misfolding and amyloidogenesis

NP-induced protein misfolding was revealed using circular dichroism (CD) spectroscopy on HSA. Exposure to nPS at concentrations up to 100 µg ml^−1^ resulted in severe dose- and size-dependent shifts in the secondary structure of HSA towards a more β-sheet-rich conformation. Specifically, 30 nm nPS, at the maximum tested concentration, decreased the α-helix content of HSA from approximately 61% in the control environment to only 25%. This loss of native helical structure was accompanied by a significant increase in β-sheet and random coil [44].

Likewise, as mentioned above, NPs induce the misfolding of ApoA-1 [45]. ApoA-1 is a primarily α-helical protein that functions as a homotetramer in lipid metabolism. As the main component in high-density lipoprotein, often referred to as ‘good cholesterol’, ApoA-1 has anti-inflammatory properties and is required for reverse cholesterol transport, whereby excess cholesterol is transported to the liver for its eventual removal from the body [52]. Importantly, ApoA-1 is highly abundant in the protein corona that forms around NPs and, upon adsorption, experiences profound conformational changes. Using high-resolution techniques, including two-dimensional infrared spectroscopy, ApoA-1 was found to form amyloid fibrils at the nPS interface. From a health perspective, it is noteworthy that ApoA-1 amyloidogenesis is associated with atherosclerosis and exacerbates the metabolic imbalances associated with diabetes [45,52].

Human Ubq also misfolds upon association with NPs. Specifically, nuclear magnetic resonance (NMR) spectroscopy studies revealed that Ubq interacts directly with nPS primarily through the C-terminal tail and the protein surface comprising the last three β-strands. Importantly, NMR analysis of the backbone nuclei confirmed global differences in chemical shifts across the entire protein structure, demonstrating significant misfolding of Ubq via interaction with nPS. This is consistent with observations from CD, which also revealed wholesale alteration of protein structure and the loss of helical elements following NP exposure [43].

MNPs, particularly nPS, physically interact with Alzheimer's disease-associated A β peptides, resulting in accelerated A β aggregation kinetics and pathology [53] (figure 1D). The hydrophobic surfaces of the plastic particles probably promote peptide adsorption. This effectively concentrates A β, thereby accelerating the rate-limiting nucleation phase of both A β40 and A β42 peptide aggregation. This surface interaction has been shown to induce conformational changes in A β secondary structure [53] and leads to increased A β aggregation in human neuroblastoma cells [53] and in mouse models of Alzheimer's disease [39,54].

Together, these findings indicate that MNPs promote disease-associated protein misfolding, while also disrupting the folding of protective proteins, such as ApoA-1, and crucial components of the proteostasis network, including Ubq.

## 4. The impact of nanoplastic on the proteostasis machinery

### (a) ubiquitin–proteasome system and protein degradation

As described above, Ubq undergoes profound conformational changes upon association with nPS in HeLa cells [43]. This disrupts the function of the Ubq–proteasome system (UPS) in HeLa cells, which, when exposed to nPS, was found to have a measurable reduction of global ubiquitination, compared with untreated cells [43]. This was similar to what was observed in a human bronchial epithelial cell line (BEAS-2B) [55]. By contrast, acute exposure of mice to NPs resulted in a dose-dependent increase in overall protein ubiquitination in the lung [55]. Thus, it appears that NPs may interfere with proteostasis in two ways, depending on the context—either by activating the UPS system, perhaps as a cytoprotective response to a generally increased load of misfolded protein, or by directly triggering the misfolding of Ubq itself, leading in turn to impaired UPS function.

### (b) Endoplasmic reticulum stress and unfolded protein response activation

Exposure to nPS induces robust endoplasmic reticulum (ER) stress, driving the acute activation of the unfolded protein response (UPR) in mammalian systems. Specifically, in the lungs of mice, the levels of key UPR proteins, including PERK, eIF2 α, IRE1 α, cleaved ATF6 α, CHOP and GRP78, all increased in response to nPS in a dose-dependent manner [55]. This was similar to findings from human colon cells, where eIF2 α and IRE1 α increased in a manner dependent on MP uptake [56]. ER-associated degradation (ERAD) is also impacted, as observed by an increase in the ERAD-associated E3 Ubq ligases HRD1 and CHIP. Specifically, these proteins were detected in mouse lungs and in tissue culture upon nPS exposure [55]. The simultaneous activation of ERAD and all three major branches of UPR suggest that nPS causes significant protein-folding stress in the ER.

### (c) Heat-shock response and oxidative stress

In human neural stem cells, the cytosolic hsp90 α and the small heat-shock protein hsp27 were reportedly upregulated in response to nPS exposure, whereas hsp70 expression was inhibited [49]. Similar findings were obtained upon exposure of aquatic insects to nPS or tyre rubber [57]. Because NP exposure also activates the expression of antioxidant genes [49,57], it is possible that nPS increases the load of misfolded protein via a mechanism that involves oxidative damage.

## 5. Concluding remarks

The widespread use of plastics for both personal and industrial applications has resulted in significant amounts of MNPs entering the air, waterways and food supply. Direct human exposure occurs primarily through oral ingestion and inhalation, with sources ranging from packaged beverages and synthetic textiles to tyre-derived crumb rubber. Once internalized, MNPs cause oxidative damage, impair macrophage function and trigger inflammatory responses. Notably, within the cellular milieu, proteins physically interact with MNPs, forming a corona that interferes with native protein structure. This interaction promotes the formation of rigid β-sheets and triggers amyloidogenesis in a manner that may pose a risk for neurodegenerative disease. Collectively, the findings highlighted in this review support the hypothesis that MNPs strain the natural ability of cells and organisms to maintain proteins and underscore how important it is to understand the mechanism by which this occurs.

## Data Availability

This article has no additional data.

## Theme

One contribution of 20 to a Theo Murphy meeting issue ‘ProteostaSys: a systems view of proteostasis'.
